# Rhei Undulati Rhizoma attenuates memory decline and reduces amyloid-β induced neuritic dystrophy in 5xFAD mouse

**DOI:** 10.1186/s13020-024-00966-2

**Published:** 2024-07-04

**Authors:** Seungmin Lee, In Gyoung Ju, Hyeyoon Eo, Jin Hee Kim, Yujin Choi, Myung Sook Oh

**Affiliations:** 1 Department of Biomedical and Pharmaceutical Sciences, Graduate School, Kyung Hee University, 26, Kyungheedae-Ro, Dongdaemun-Gu, Seoul, 02447 Republic of Korea; 2 Department of Oriental Pharmaceutical Science and Kyung Hee East-West Pharmaceutical Research Institute, College of Pharmacy, Kyung Hee University, 26, Kyungheedae-Ro, Dongdaemun-Gu, Seoul, 02447 Republic of Korea; 3 Department of Integrated Drug Development and Natural Products, Graduate School, Kyung Hee University, 26, Kyungheedae-Ro, Dongdaemun-Gu, Seoul, 02447 Republic of Korea

**Keywords:** Rhei Undulati Rhizoma, Amyloid-β, Dystrophic neurites, β-Site amyloid precursor protein cleaving enzyme-1, Tau hyperphosphorylation

## Abstract

**Background:**

Alzheimer's disease (AD) is a common type of dementia characterized by amyloid-β (Aβ) accumulation, lysosomal dysfunction, and tau hyperphosphorylation, leading to neurite dystrophy and memory loss. This study aimed to investigate whether Rhei Undulati Rhizoma (RUR), which has been reported to have anti-neuroinflammatory effect, attenuates Aβ-induced memory impairment, neuritic dystrophy, and tau hyperphosphorylation, and to reveal its mode of action.

**Methods:**

Five-month-old 5xFAD mice received RUR (50 mg/kg) orally for 2 months. The Y-maze test was used to assess working memory. After behavioral testing, brain tissue was analyzed using thioflavin S staining, western blotting, and immunofluorescence staining to investigate the mode of action of RUR. To confirm whether RUR directly reduces Aβ aggregation, a thioflavin T assay and dot blot were performed after incubating Aβ with RUR.

**Results:**

RUR administration attenuated the Aβ-induced memory impairment in 5xFAD mice. Furthermore, decreased accumulation of Aβ was observed in the hippocampus of the RUR-treated 5xFAD group compare to the vehicle-treated 5xFAD group. Moreover, RUR reduced the dystrophic neurites (DNs) that accumulate impaired endolysosomal organelles around Aβ. In particular, RUR treatment downregulated the expression of β-site amyloid precursor protein cleaving enzyme 1 and the hyperphosphorylation of tau within DNs. Additionally, RUR directly suppressed the aggregation of Aβ, and eliminated Aβ oligomers in vitro.

**Conclusions:**

This study showed that RUR could attenuate Aβ-induced pathology and directly regulate the aggregation of Aβ. These results suggest that RUR could be an efficient material for AD treatment through Aβ regulation.

**Graphical Abstract:**

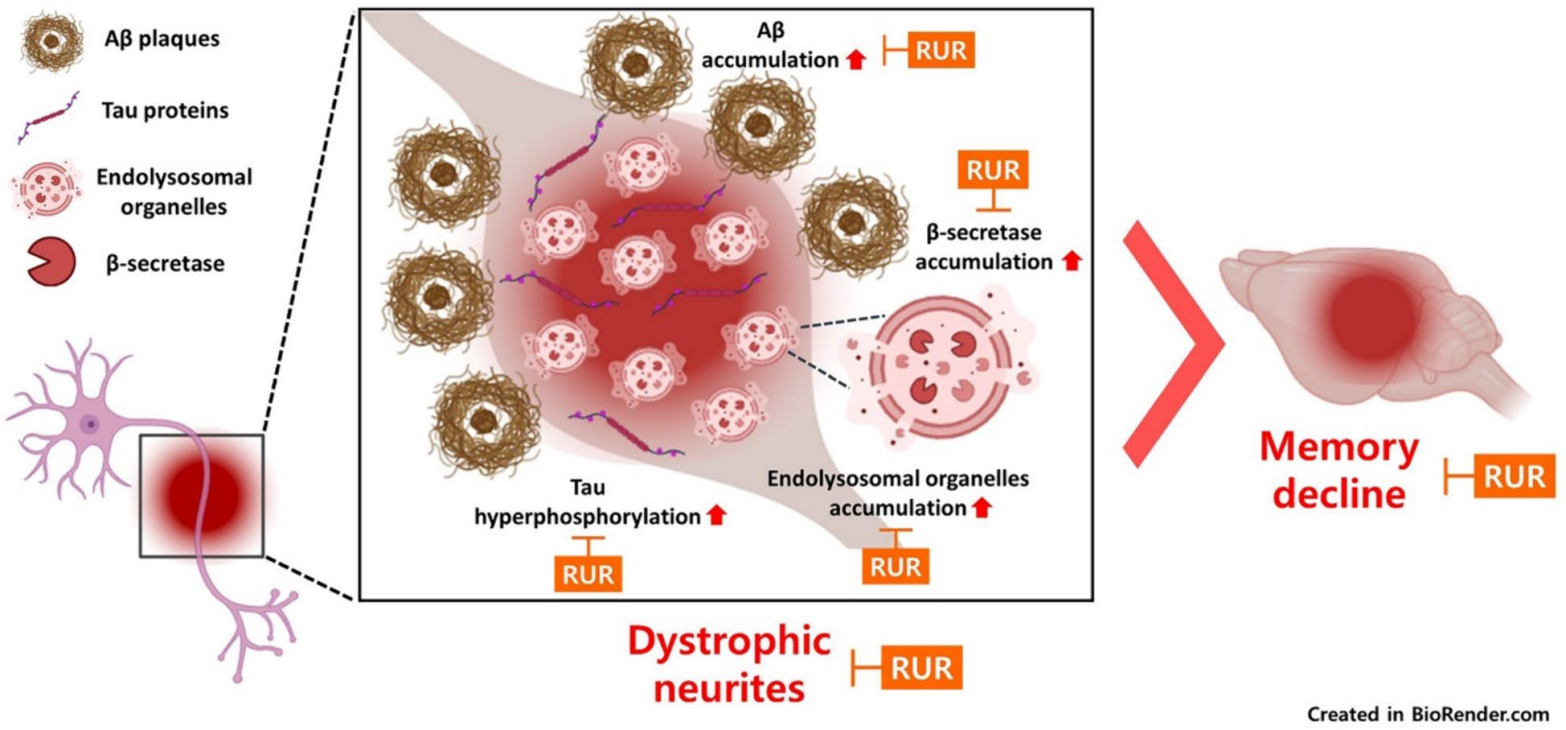

**Supplementary Information:**

The online version contains supplementary material available at 10.1186/s13020-024-00966-2.

## Introduction

Alzheimer’s disease (AD), a neurodegenerative disorder, is the most common type of dementia and is characterized by memory loss, cognitive difficulties, and aggressive behavior [1]. In AD, brain volume decreases due to neuronal cell death and in severe cases, brain atrophy is observed [2]. Misfolded proteins, such as amyloid-β (Aβ) and tau accumulate in the brain of patients with AD, and these factors induce neuronal cell death, neuroinflammation, and synaptic loss [3]. Among the various hypotheses in the etiology of AD, the Aβ cascade hypothesis has been widely studied as a key pathology in AD [4].

In the amyloidogenic pathway, β-site amyloid precursor protein (APP) cleaving enzyme 1 (BACE1) cleaves the β-site of APP and generates C-terminal fragment (CTF)-β, instead of the α-secretase which cleaves the α-site of APP [5]. This fragment is then further cleaved by γ-secretase to generate Aβ [6]. Sequential cleavage of APP by β-secretase and γ-secretase results in the aggregation of Aβ in the brain [7]. The monomeric Aβ generated from the amyloid processing pathway aggregates into various forms such as protofibrils, oligomers, and plaques [8]. The aggregated forms of Aβ cause neurotoxicity and induce neuronal cell death in the hippocampus which plays a critical brain region in terms of learning and memory formation [9]. To protect neuronal cells from neurotoxic Aβ, the clearance mechanism, such as the endolysosomal pathway, is activated [10].

Lysosomes are membrane-bound vesicles containing various enzymes, including proteases, phosphatases, and nucleases, which are known as cellular organelles responsible for the degradation of cellular molecules, such as proteins, lipids, and carbohydrates, through endocytosis [11]. Lysosomes also play an important role in homeostasis, cellular signaling, and metabolism [12]. Unfortunately, individuals with AD exhibit endolysosomal dysfunction [13]. Dysfunctional endolysosomal organelles accumulate in dystrophic neurites (DNs) around the Aβ deposit [14], and hyperphosphorylated tau is also observed within the DNs [15]. Accumulated DNs induce focal axonal swellings that interfere with the trafficking and decomposition pathways of enzymes involved in Aβ generation such as β-secretase [16]. Furthermore, recent studies have shown that DNs are correlated with the severity of AD [17]. Therefore, downregulation of Aβ aggregation and alleviation of neuritic dystrophy could be a potential therapeutic strategy for the treatment of AD.

Rhei Undulati Rhizoma (RUR), an herbal medicine belonging to the family Polygonaceae, have traditionally been used to treat constipation, ulcers, and jaundice in the East Asia [18]. Furthermore, recent studies have reported that RUR has various pharmacological activities such as anti-inflammatory, antioxidant, and anti-neuroinflammatory effects [19–21]. Furthermore, rhapontigenin in stilbene glucosides isolated from RUR has a neuroprotective effect against Aβ-induced neurotoxicity [22]. Based on previous reports, we hypothesized that RUR attenuates AD pathology.

In this study, we evaluated the effect of RUR on the pathological characteristics of AD in the 5xFAD mouse. We administered RUR orally to 5xFAD transgenic mice and performed a behavior test to investigate the effect of RUR on Aβ-induced memory loss. Furthermore, we measured the effects of RUR on Aβ accumulation, neuritic dystrophy, and tau hyperphosphorylation in the hippocampus of the brain of 5xFAD mice. Furthermore, we performed a thioflavin T (Th T) fluorescence assay to evaluate the anti-aggregation effect of RUR on β-sheet rich forms of Aβ.

## Materials and methods

### Materials

Horseradish peroxidase (HRP) conjugated mouse anti-β-actin antibody was purchased from Santa Cruz Biotechnology (Temecula, CA, USA). Rabbit anti-glyceraldehyde-3-phosphate dehydrogenase (GAPDH) antibody, rabbit-anti-Protein kinase B (Akt) antibody, rabbit anti-phospho-Akt (serine 473) antibody, rabbit anti-Glycogen synthase kinase-3 (GSK-3β) antibody, rabbit anti-phospho-GSK-3β (serine 9), and rabbit anti-lysosomal-associated membrane protein 1 (LAMP1) antibody were purchased from Cell Signaling Technology (Danvers, MA, USA). Mouse anti-β-Amyloid antibody (6E10) was purchased from BioLegend (San Diego, CA, USA). Aβ_1-42_ peptide was purchased from AnaSpec (Fremont, CA, USA). Skim milk was purchased from BD Transduction Laboratories (Franklin Lakes, NJ, USA). Mouse anti-BACE1 antibody and polyvinylidene difluoride (PVDF) was purchased from Millipore (Burlington, MA, USA). Normal horse serum and anti-fade fluorescent mounting medium containing 4′,6-diamidino-2-phenylindole were purchased from Vector Laboratories (Burlingame, CA, USA). Anti-mouse and anti-rabbit HRP secondary antibodies were purchased from Enzo Life Sciences, Inc. (Farmingdale, NY, USA). Tetramethylethylenediamine, protein assay reagent, acrylamide, enhanced chemiluminescence (ECL) reagent, protein standards dual color, and Tween 20 were purchased from Bio-Rad Laboratories (Hercules, CA, USA). Rabit-anti-CTF antibody, mouse anti-AT8 (phosphor-tau, serine 202/threonine 205) antibody, rabbit anti-oligomer antibody (A11), goat anti-rabbit Alexa 488, goat anti-mouse Alexa 594, and protease/phosphatase inhibitor cocktail were purchased from Thermo Fisher Scientific (Waltham, MA, USA). Nordihydroguaiaretic acid (NDGA), Th T, thioflavin S (Th S), and all the other reagents were purchased from Sigma-Aldrich (St. Louis, MO, USA), unless otherwise noted.

### Preparation of RUR extract

RUR was purchased from the Kwangmyoungdang Medicinal Herbs (Naemomedah, Ulsan, Republic of Korea). RUR, the voucher specimen (BON19012401), was deposited in the herbarium of the College of Pharmacy at Kyung Hee University (Seoul, Republic of Korea). The dried rhizomes were extracted with 70% ethanol on rocking shaker for 24 h at room temperature. The extract was then filtered and lyophilized to obtain a powder (yield: 28.50%). The extract was resuspended in an appropriate vehicle before use. Extract of RUR was standardized the contents of rhapontin and rhapontigenin, the principal compounds of RUR that are known to suppress neuroinflammation and attenuate Aβ/tau-related AD pathological features, using an ultra performance liquid chromatography-photodiode array analysis [19, 23]

### Animals and administration

We purchased 5xFAD (B6SJL-Tg(APPSwFlLon, PSEN1*M146L*L286V)6799Vas/Mmjax) mouse from the Jackson Laboratory (Bar Harbor, ME, USA). 5xFAD mutations include APP KM670/671NL (Swedish), APPI716V (Florida), APPV717I (London), PSEN1 M146L, and PSEN1 L286V, resulting in early and aggressive Aβ accumulation related to memory deficits [24]. Five-month-old male and female wild-type (WT) and 5xFAD mouse were used in the experiments. Mouse were divided into three groups: WT (n = 21), 5xFAD (n = 11), and 5xFAD + RUR at 50 mg/kg (n = 11). RUR at 50 mg/kg was dissolved in 2% tween 80 and orally administered using a Zonde needle to 5xFAD mouse for 2 months. The mouse were housed in plastic cages with constant temperature (23 ± 1 °C), humidity (50 ± 10%), and a 12 h light/dark cycle and free access to food and water. In this study, all animal studies were performed in accordance with the ‘Guide for the Care and Use of Laboratory Animals, 8th edition’ (National Institutes of Health, 2011).

### Y-maze test

The Y-maze test was performed using a Y-shaped maze consisting of three arms (40 cm × 3 cm × 12 cm walls) to assess working memory [25]. The mouse was placed in the center of the Y-maze and allowed to explore each arm labeled A, B, or C. Arm entries and sequences were recorded for 8 min. Alternation behavior was defined as consecutive entries in three different arms without repetition: ABC, BCA, or CBA. The percentage of spontaneous alternations was calculated using the following equations: (number of alternations/total number of arm entries − 2) × 100.

### Brain tissue preparation

For tissue analysis, the mice were anesthetized and transcardially perfused with 0.05 M phosphate-buffered saline (PBS). After perfusion was complete, the mouse was fixed with 4% para-formaldehyde in 0.1 M phosphate buffer. The dissected brains were then post-fixed overnight at 4 °C, and immersed in 30% sucrose dissolved in PBS for cryoprotection. Serial 25 μm thick coronal sections were cut on a freezing sliding microtome (Leica Microsystems Inc., Nussloch, Germany) and stored in a cryoprotectant (25% ethylene glycol, 25% glycerol, and 0.05 M phosphate buffer) at 4 °C until use.

### Thioflavin S (Th S) staining

For staining Aβ plaques, we used Th S, which interacts with the β-sheet structure of amyloid plaques and commonly visualizes the region of Aβ plaques in the mouse brain [26]. The free-floating sections were washed with PBS and mounted on adhesion microscope slides. The slides were incubated in the dark with 0.5% Th S for 20 min, rinsed with 50%, 70%, 90%, and 100% ethanol for 2 min each, and covered with mounting medium.

### Immunofluorescence staining

For immunofluorescence staining, free-floating sections of mouse brains were rinsed in PBS and incubated for 1 h in a blocking solution containing 3% normal goat or horse serum, 2% bovine serum albumin, and 0.3% Triton X-100 at RT. After the blocking step, the sections were incubated with primary antibodies overnight at 4°C. For visualization, they were subsequently incubated with secondary antibody for 1 h at RT. The sections were mounted and the cover-slipped using an anti-fade mounting medium containing 4′,6-diamidino-2-phenylindole. Fluorescent images were captured using a K1-Fluo confocal microscope (Nanoscope Systems, Daejeon, Republic of Korea).

### Western blot

Western blot was performed as previously described [27]. The hippocampal regions of mouse brains were dissected in the vehicle-treated WT group, vehicle-treated 5xFAD group, and RUR-treated 5xFAD group. Then, the hippocampus was homogenized in radio-immunoprecipitation assay lysis buffer (RIPA) containing protease/phosphatase inhibitors. The protein amount of the sample buffer was equalized to 30 μg using the Bradford assay. Protein samples were separated by sodium dodecyl sulfate–polyacrylamide gel electrophoresis, and transferred to PVDF membranes. Next, the membranes were incubated with blocking solution containing 5% bovine serum albumin or skim milk in 0.1% Tween 20 in tris-buffered saline for 1 h at RT, and reacted with primary antibodies overnight at 4 °C. After that, they were incubated with HRP-conjugated secondary antibodies for 1 h at RT. The immunoreactive bands on the membrane were detected by the ECL reagent and visualized using ChemiDocXRS + imaging system (Bio-Rad Laboratories). The quantification of band intensity was performed using the ImageJ software (Bethesda, MD, USA).

### Aβ enzyme-linked immunosorbent assay (ELISA)

Aβ_1-42_ and Aβ_1-40_ ELISA was performed using fluorescent-based kit (Invitrogen, Camarillo, CA, USA) and appropriate Aβ_1-42_ and Aβ_1-40_ standards based on the product guideline, respectively. To obtain soluble fraction, right half of hippocampus was homogenized in RIPA buffer containing protease/phosphatase inhibitor and incubated on ice for 2h. After centrifugation at 15,000 rpm for 20 min, the supernatant (RIPA fraction) was collected and used as soluble proteins. The pellets were subsequently dissolved in 70% formic acid and incubated on ice for 2 h. After centrifugation at 15,000 rpm for 20 min, the supernatant (formic acid fraction) was neutralized by Tris base buffer and used as insoluble proteins [28]. Protein concentrations of soluble and insoluble protein samples were determined using the Bradford protein assay and Lowry protein assay, respectively.

### Th T assay

Th T is commonly used to measure Aβ fibril aggregation and enhanced fluorescence emission when bound to the β-sheet of Aβ fibrils [26]. Aβ_1-42_ monomer (5 μL of 100 μM in dimethyl sulfoxide) was incubated with PBS or RUR (0.3, 3, or 30 μg/mL) for 48 h at 37 °C. Then, 150 μL of 5 μM Th T solution diluted with 50 mM glycine-sodium hydroxide at pH 8.5 was added and incubated for 30 min at RT. Th T fluorescence was measured at 520 nm with excitation at 470 nm by FLUOstar Omega multimode microplate reader (BMG LABTECH GmbH, Ortenberg, Germany).

### Dot blot

The Aβ_1-42_ monomer (25 μM in DMEM/F-12 1:1 mixture) was incubated with PBS or RUR (0.3, 3, or 30 μg/mL) for 24 h at 4°C to measure the effect of RUR on Aβ_1-42_ oligomerization. Furthermore, the Aβ_1-42_ oligomer (25 μM in DMEM/F-12 1:1 Mixture) was incubated with PBS or RUR (0.3, 3, or 30 μg/mL) for 3 h at 4 °C to measure the effect of RUR on Aβ_1-42_ oligomer degradation. Then, 2 μL of each sample was spotted on PVDF membranes. The membrane was reacted with primary antibodies overnight at 4 °C. The membranes were incubated with HRP-conjugated secondary antibodies for 1 h at RT. The immunoreactive bands on the membrane were detected using an ECL reagent and visualized using a ChemiDocXRS + imaging system (Bio-Rad Laboratories). The quantification of the band intensity was performed using ImageJ software (Bethesda, MD, USA).

### Statistical analysis

All statistical parameters were calculated using GraphPad Prism version 8.0 software (Graphpad Software, San Diego, CA, USA). Values were expressed as the mean ± standard error of the mean, and analyzed using one-way analysis of variance (ANOVA), followed by Dunnett’s post hoc test or an unpaired Student's t-test. Differences with a *p* value less than 0.05 were considered statistically significant.

## Results

### Effect of RUR administration on memory decline in the 5xFAD mouse

**Fig. 1:**
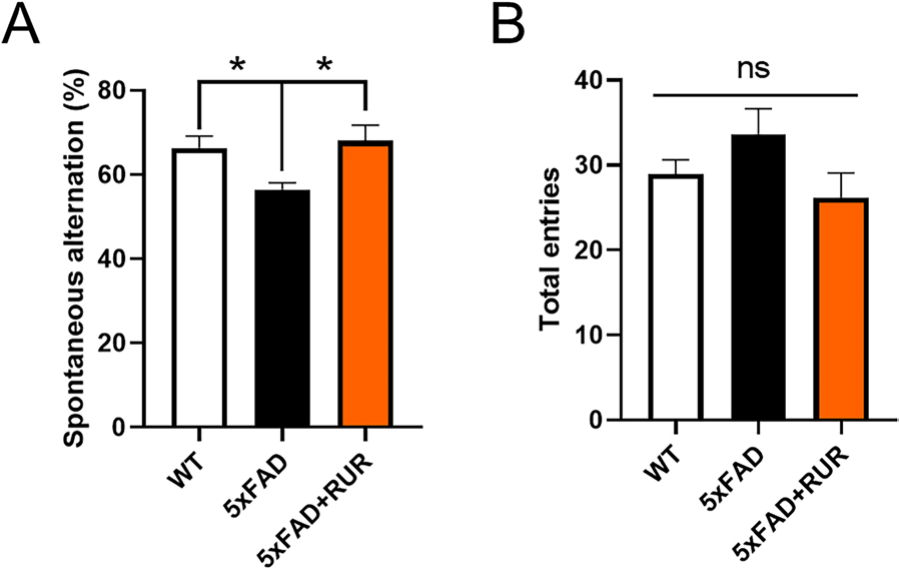
Effects of Rhei Undulati Rhizoma (RUR) on memory decline in the 5xFAD mouse. Five-month-old WT and 5xFAD mice were administered vehicle or RUR (50 mg/kg) for 2 months. In the Y-maze test, spontaneous alternations (**A**) and total entries (**B**) were measured. The statistical analyses were performed using one-way analysis of variance (ANOVA), followed by Dunnett’s post hoc test. *p < 0.05 vs. 5xFAD group

The 5xFAD mice, a transgenic mouse well known as an AD model, are characterized by increased Aβ accumulation in the brain. Furthermore, in this model, memory impairment caused by Aβ accumulation is observed [24]. To identify whether RUR alleviates memory loss in 5xFAD mice, we administered RUR to 5-month-old 5xFAD mice for 2 months and performed a Y-maze test to assess working memory [29]. The total number of entries did not differ between the groups. The 5xFAD group showed a significantly lower percentage of spontaneous alternation (%) than the WT group. However, treatment with 50 mg/kg RUR markedly increased the percentage of spontaneous alternations (Fig. 1). Additionally, we performed MWM to assess the effect of RUR on spatial memory [30]. On the 5th day of training, the vehicle-treated 5xFAD group showed slower escape latency than the vehicle-treated WT group. However, the RUR-treated 5xFAD group had exhibited faster escape latency compared to the vehicle-treated 5xFAD group. Moreover, the time spent in the target quadrant on the probe trial, was significantly reduced in the vehicle-treated 5xFAD group compared to the vehicle-treated WT group, whereas this level was elevated in the RUR-treated 5xFAD group (Supplementary Figure S1).

### Effect of RUR on Aβ accumulation in the brain of the 5xFAD mouse

**Fig. 2:**
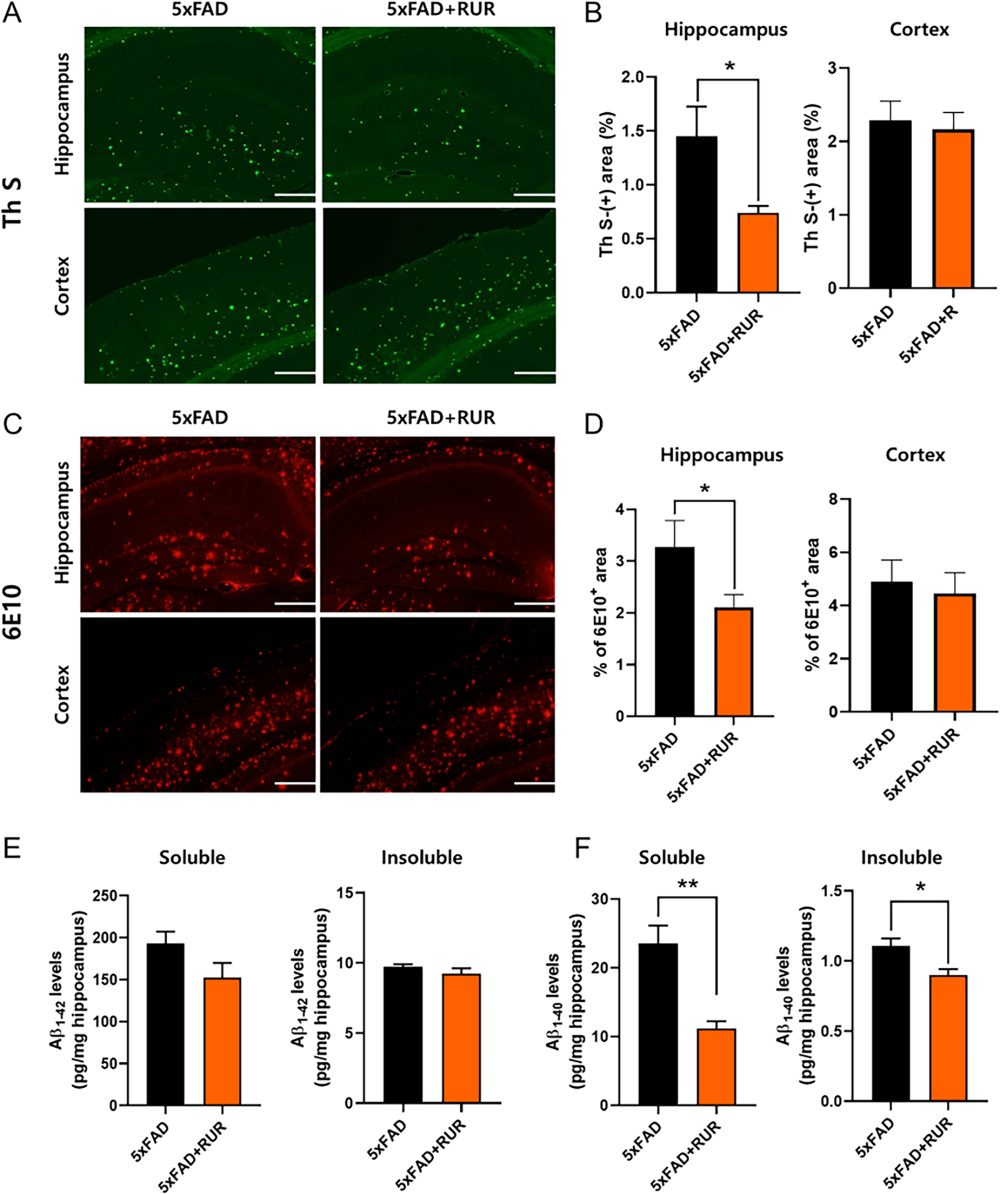
Effect of RUR on Aβ accumulation in the brain of the 5xFAD mouse. Representative photomicrographs and quantifications for Th S staining (**A**, **B**) and 6E10 immunopositive areas (**C**, **D**) are shown in the hippocampus and cortex (n = 5 − 6 per group). Analysis of soluble and insoluble levels of Aβ_1-42_ and Aβ_1-40_ from the hippocampus of the mouse using ELISA kits (n = 5 − 6 per group). The statistical analyses were performed by unpaired Student’s *t* test. *p < 0.05 and **p < 0.01 vs. 5xFAD group. Scale bar = 200 μm. Aβ, amyloid-β; Th S, thioflavin S; 6E10, anti-Aβ antibody

To determine whether RUR could suppress the accumulation of Aβ, we stained the deposition of Aβ within the hippocampus and cortex using Th S staining and immunohistochemical analysis using 6E10. In Th S staining, RUR markedly reduced β-sheet-rich amyloid plaques in the hippocampus (Fig. 2A, B). Furthermore, RUR-treated 5xFAD mice had a significant decrease in 6E10-positive Aβ protein levels compared to vehicle-treated 5xFAD mice (Fig. 2C, D). However, in the cortex, no differences were identified between the two groups in either assay. Moreover, we measured Aβ_1-42_ and Aβ_1-40_ levels in the hippocampus of 5xFAD mouse using ELISA kit. RUR administration reduced the soluble form of Aβ_1-42_ and Aβ_1-40_ levels and insoluble form of Aβ_1-40_ level in the hippocampus (Fig. 2E, F).

**Fig. 3:**
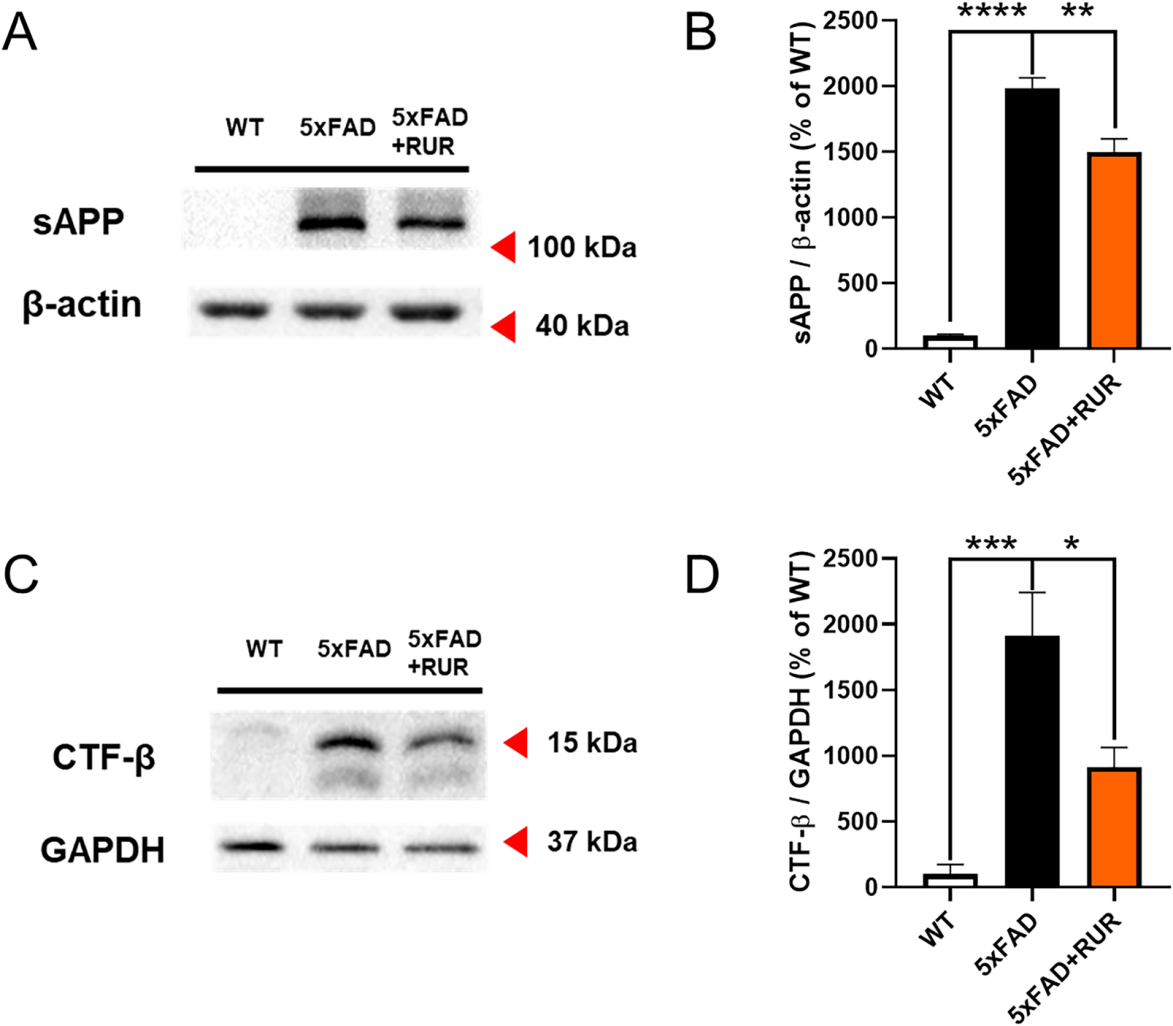
Effect of RUR on derivatives of APP by cleavage in the hippocampus. The representative band image (**A**) and quantification (**B**) of sAPP are shown (n = 4 − 5 per group). The protein level of sAPP was normalized to β-actin. The representative band image (**C**) and quantification (**D**) of CTF- β are shown (n = 4 − 5 per group). The protein level of CTF- β was normalized to GAPDH. The statistical analyses were performed using ANOVA, followed by Dunnett’s post hoc test. ****p < 0.0001, ***p < 0.001, **p < 0.01, and *p < 0.05 vs. 5xFAD group. *APP* amyloid precursor protein, *sAPP* soluble APP, *CTF-β* c-terminal fragment –β, *GAPDH* glyceraldehyde-3-phosphate dehydrogenase

We also performed a western blot analysis to measure the protein expression levels of APP derivatives after cleavage. The protein level of soluble APP (sAPP) was higher in 5xFAD mice than in WT mice, while RUR remarkably downregulated the expression of the sAPP protein (Fig. 3A, B). Furthermore, we measured the CTF-β produced when APP is cleaved by β-secretase. We first measured the protein levels of CTF-β through western blotting. CTF-β protein was overexpressed in the vehicle-treated 5xFAD group compared to the vehicle-treated WT group. However, RUR treatment markedly reduced both fragments in a manner similar to that of sAPP (Fig. 3C, D).

### Effect of RUR administration on the accumulation of DNs in the hippocampus of the 5xFAD mouse

**Fig. 4:**
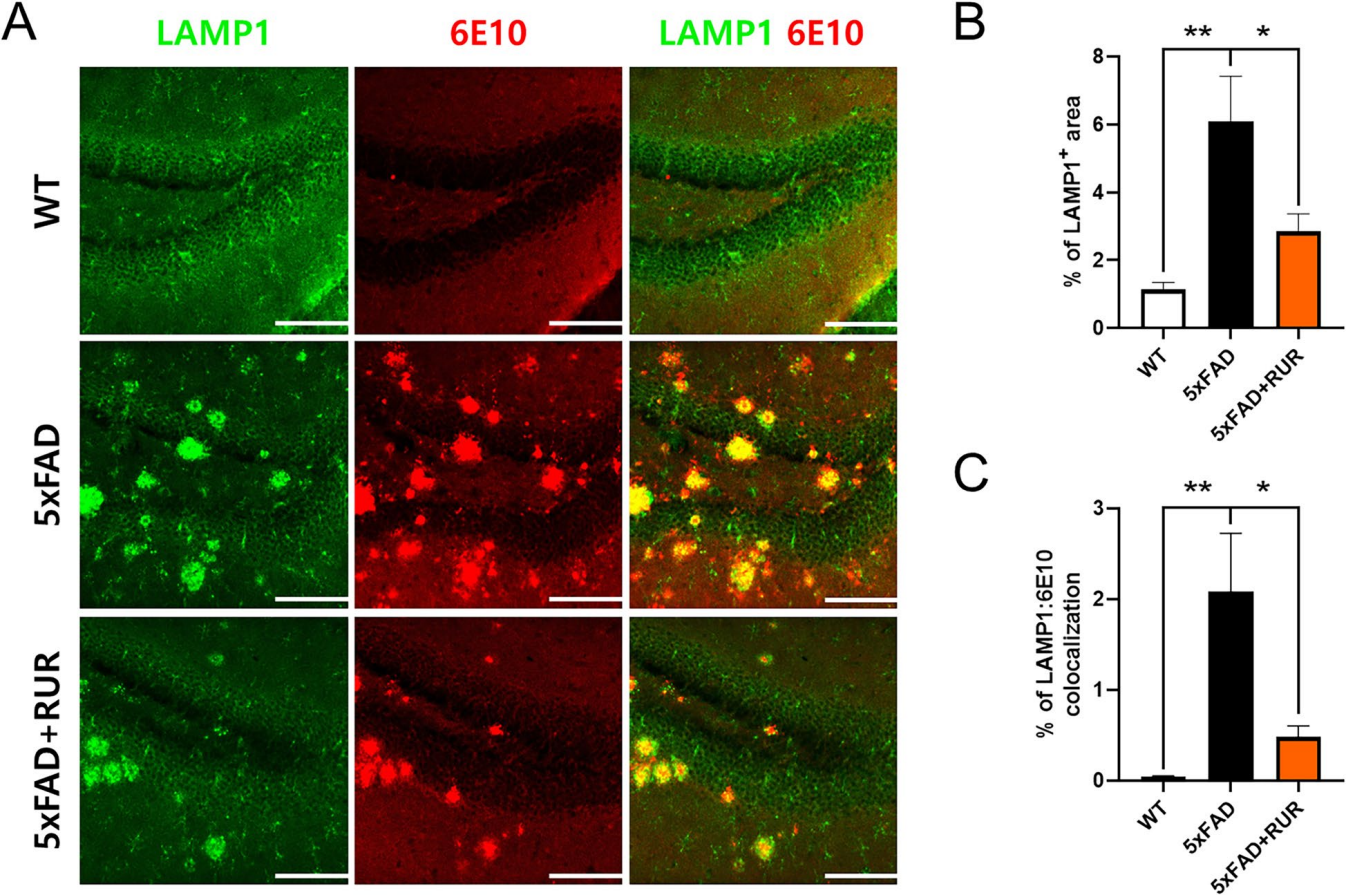
Effect of RUR administration on the accumulation of DNs in the hippocampus. Representative photomicrographs of LAMP1 (green) and 6E10 (red) immunopositive areas in the hippocampus are shown (n = 5 − 6 per group) (**A**). The quantification of the LAMP1-positive area was measured by ImageJ (**B**). The percentage of colocalization (LAMP1:6E10) was measured using a colocalization finder from ImageJ (**C**). The statistical analyses were performed using ANOVA, followed by Dunnett’s post hoc test. **p < 0.01 and *p < 0.05 vs. 5xFAD group. Scale bar = 200 μm. *LAMP1* lysosomal-associated membrane protein 1

To examine whether RUR could ameliorate the accumulation of DNs around the Aβ peptide, we co-stained with LAMP1 as the lysosomal marker, and 6E10 as the Aβ marker. The LAMP1-positive area was significantly larger in the vehicle-treated 5xFAD group than in the vehicle-treated WT group. In contrast, RUR treatment markedly reduced LAMP1 positive areas in the hippocampus (Fig. 4A, B). We also quantified the region of LAMP1 and 6E10 colocalization to assess whether RUR reduces the accumulated DNs around Aβ. We observed that RUR treatment reduced the co-stained regions of LAMP1 and 6E10, indicating that RUR treatment alleviated the accumulation of DNs (Fig. 4C).

### Effect of RUR administration on the expression of BACE1 within DNs in the hippocampus of the 5xFAD mouse

**Fig. 5:**
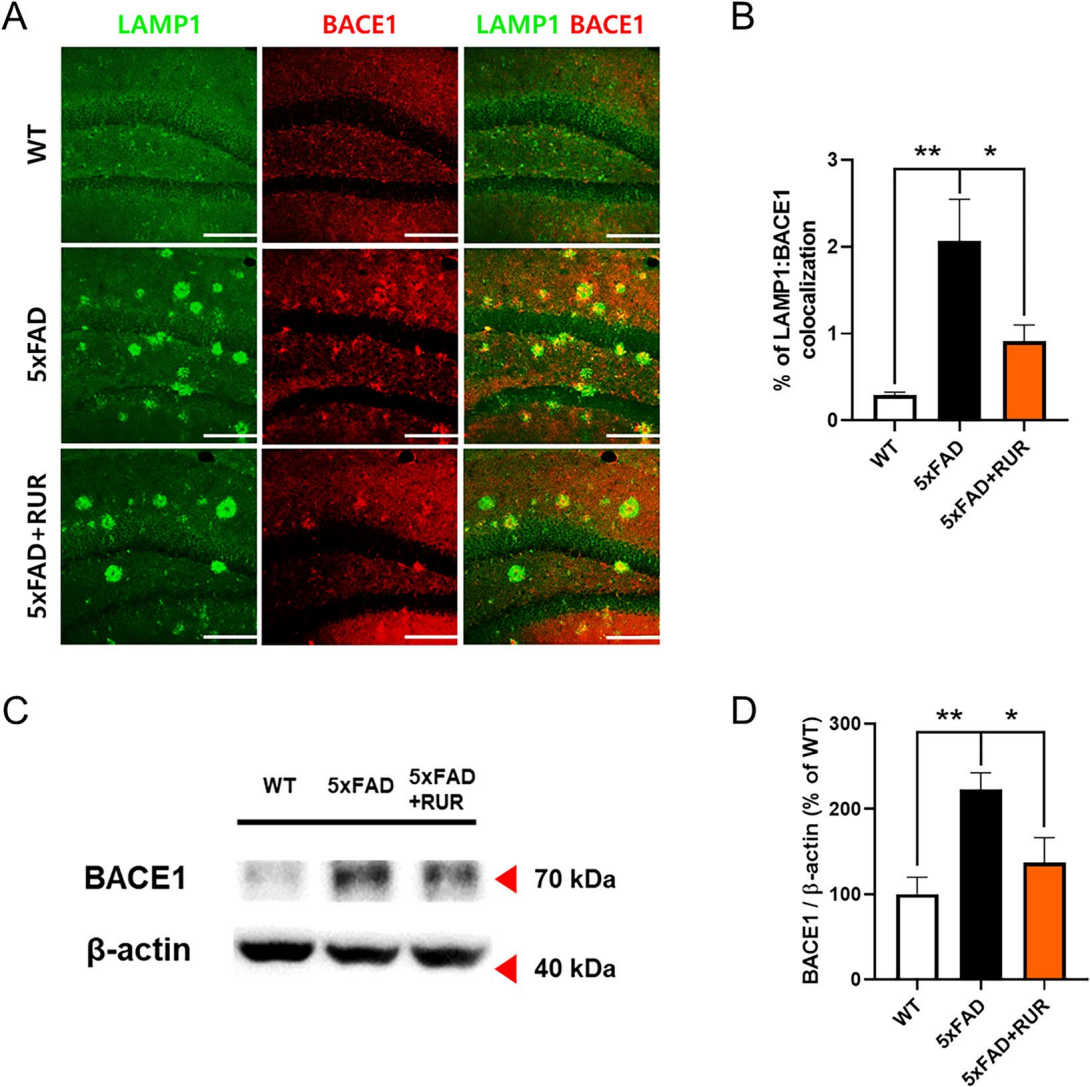
Effect of RUR administration on the expression of BACE1 within DNs in the hippocampus. Representative photomicrographs of LAMP1 (green) and BACE1 (red) immunopositive areas in the hippocampus are shown (n = 5 − 6 per group) (**A**). The percentage of colocalization (LAMP1:BACE1) was measured using a colocalization finder from ImageJ (**B**). The representative band image (**C**) and quantification (**D**) of BACE1 are shown (n = 4 − 5 per group). The protein level of BACE1 was normalized to β-actin. The statistical analyses were performed using ANOVA, followed by Dunnett’s post hoc test. **p < 0.01 and *p < 0.05 vs. 5xFAD group. Scale bar = 200 μm. *BACE1* β-site amyloid precursor protein cleaving enzyme 1

To measure whether RUR could attenuate BACE1 accumulation within DNs around Aβ burden, we measured BACE1 levels within DNs using double immunofluorescence staining for LAMP1 and BACE1 in the hippocampus. The area co-stained with LAMP1 and BACE1 was larger in the vehicle-treated 5xFAD group than in the vehicle-treated WT group, while this area was significantly reduced in the RUR-treated 5xFAD group (Fig. 5A, B). BACE1 levels in the hippocampi of 5xFAD mice were measured by western blot analysis using anti-BACE1 antibody. The expression of BACE1 was significantly upregulated in the 5xFAD group compared to that in the WT group. Furthermore, 50 mg/kg RUR markedly reduced BACE1 expression compared to the 5xFAD mouse group (Fig. 5C, D, Supplementary Figure S2).

### Effect of RUR administration on the hyperphosphorylation of tau within DNs in the hippocampus of the 5xFAD mouse

**Fig. 6:**
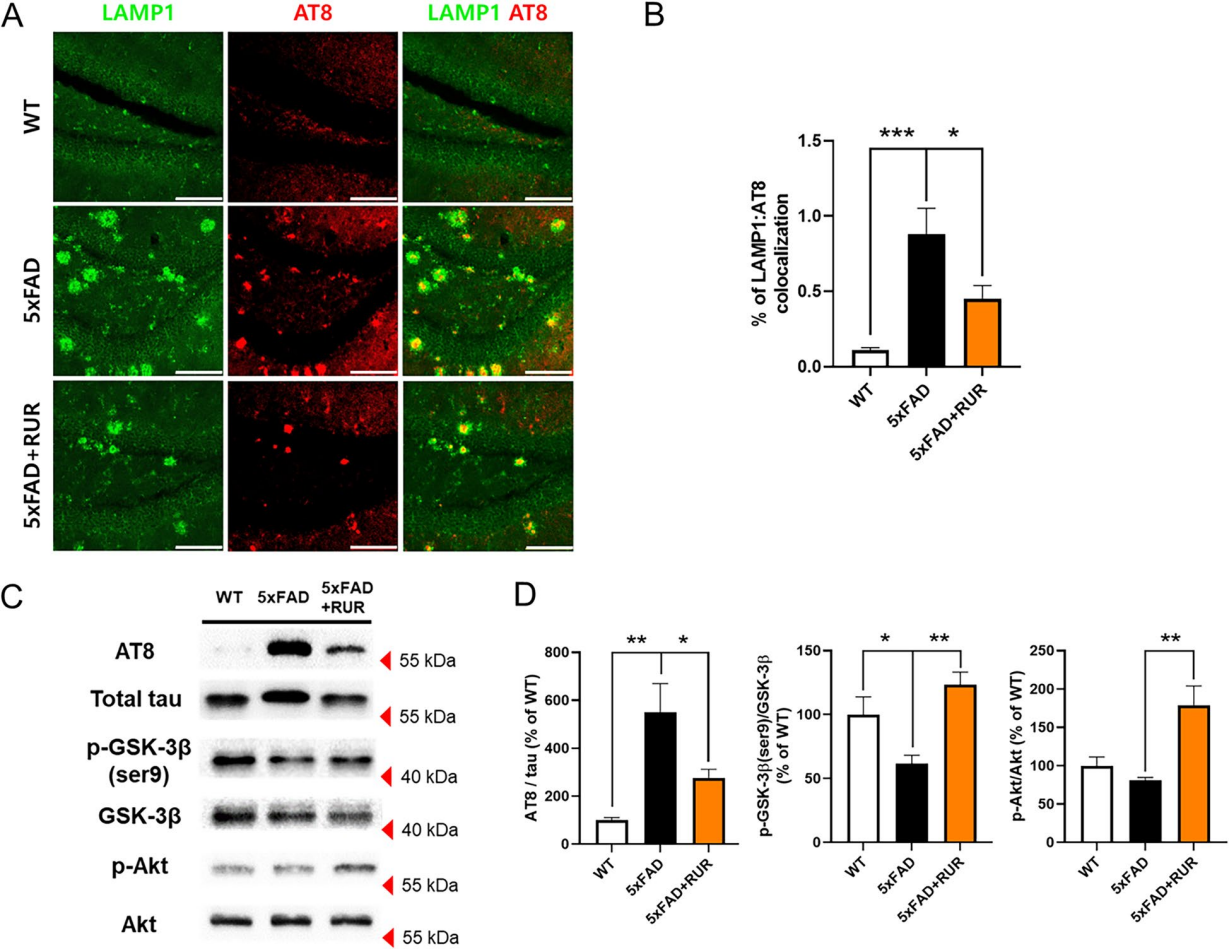
Effect of RUR administration on the hyperphosphorylation of tau within DNs in the hippocampus. Representative photomicrographs of LAMP1 (green) and AT8 (red) immunopositive areas in the hippocampus are shown (n = 5 − 6 per group) (**A**). The percentage of colocalization (LAMP1:AT8) was measured using a colocalization finder from ImageJ (**B**). The representative band (**C**) and quantification of the AT8 normalized to total tau, p-GSK-3β (ser9) normalized to total GSK-3β, and p-Akt normalized to total Akt (**D**) are shown (n = 4 − 5 per group). The statistical analyses were performed using ANOVA, followed by Dunnett’s post hoc test. ***p < 0.001, **p < 0.01 and *p < 0.05 vs. 5xFAD group. Scale bar = 200 μm

To assess whether RUR alleviated tau phosphorylation within DNs, we co-stained hippocampal sections with LAMP1 and AT8 antibodies. The co-stained region of LAMP1 and AT8 increased markedly in the vehicle-treated 5xFAD group compared to the vehicle-treated WT group. However, this area was significantly reduced in the RUR-treated 5xFAD group compared to that in the vehicle-treated 5xFAD group (Fig. 6A, B). Phosphorylated levels of tau in the hippocampus were measured by western blot analysis using anti-AT8. Similarly to the results of immunofluorescence staining, the phosphorylated ratio of tau was increased in 5xFAD mice compared to WT mice, while RUR administration reduced the ratio in the hippocampus (Fig. 6C, D). To explore whether RUR ameliorated the hyperphosphorylation of tau, we measured the phosphorylation of GSK-3β. In AD conditions, the activation of GSK-3β is increased and induces tau hyperphosphorylation [31]. However, the phosphorylation GSK-3β at the serine 9 by several kinases such as Akt, suppresses GSK-3β activation [32]. The GSK-3β phosphorylation at serine 9 was reduced in vehicle-treated 5xFAD group, compared to the vehicle treated WT group, whereas RUR treatment increased the serine 9-phosphorylated form of GSK-3β. Moreover, RUR administration increased Akt phosphorylation. Taken together, RUR downregulates GSK-3β activation by the phosphorylation of serine 9 site via Akt activation, and these results suggest that RUR treatment suppresses tau hyperphosphorylation in the hippocampus of 5xFAD mouse (Fig. 6C, D).

### Effect of RUR on Aβ aggregation in vitro

**Fig. 7:**
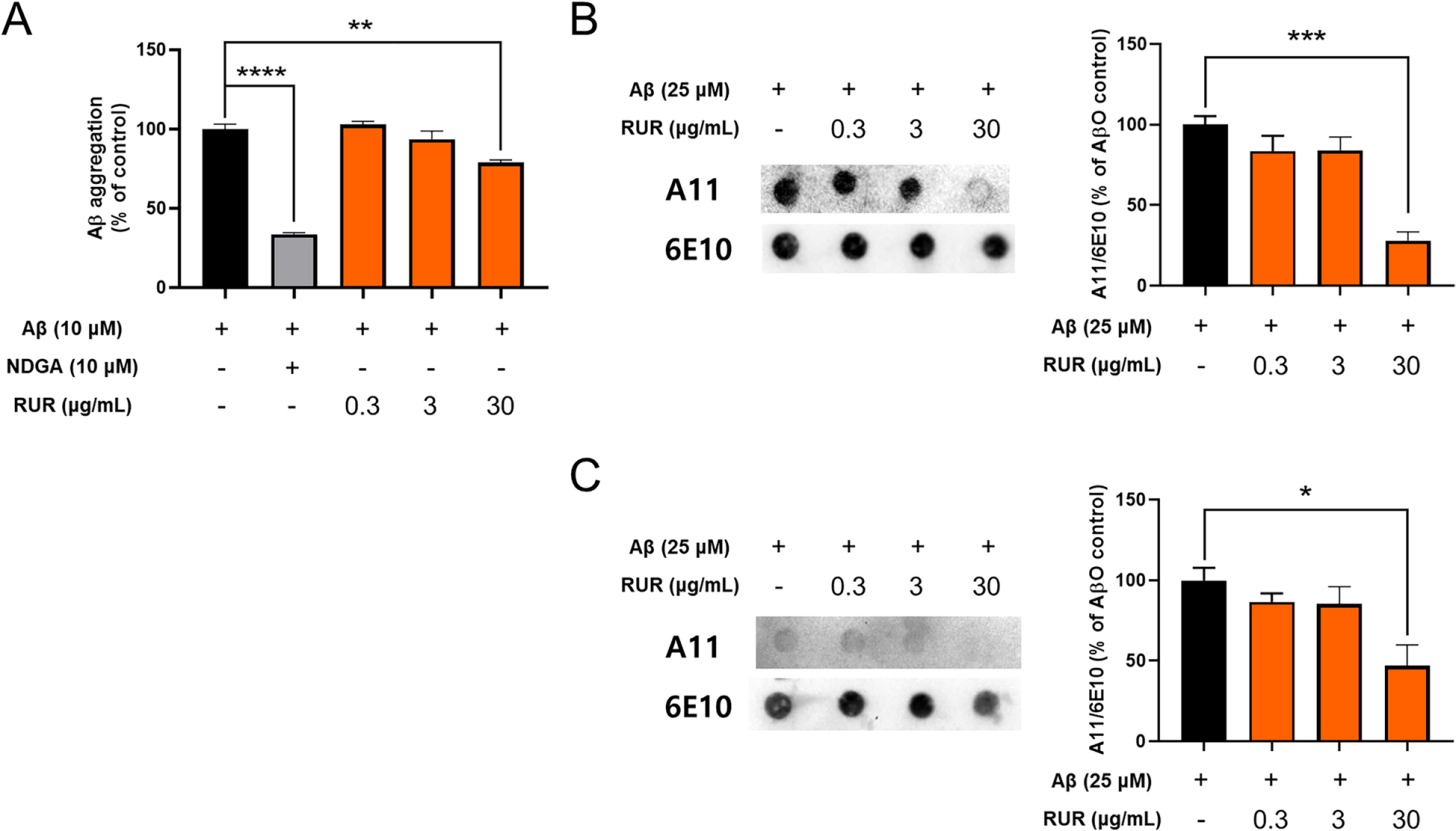
Effect of RUR on Aβ aggregation in vitro assay. A thioflavin T assay was performed to measure the effects of RUR on Aβ aggregation. Aβ_1-42_ monomer (5 μL of 100 μM) was incubated with PBS or RUR (0.3, 3, or 30 μg/mL) for 48 h at 37 °C (**A**). Dot blot analysis was performed to measure the effects of RUR on Aβ oligomerization. The Aβ_1-42_ monomer (25 μM) was incubated with PBS or RUR (0.3, 3, or 30 μg/mL) for 24 h at 4 °C (**B**). Furthermore, the Aβ_1-42_ oligomer (25 μM) was incubated with PBS or RUR (0.3, 3, or 30 μg/mL) for 3 h at 4 °C to measure the effect of RUR on Aβ_1-42_ oligomer degradation (**C**). Representative bands of A11 and 6E10 were shown (n = 3 per group). The quantification of the A11 band were normalized to the 6E10 band in dot blot analysis. The statistical analyses were performed using ANOVA, followed by Dunnett’s post hoc test. ****p < 0.0001, ***p < 0.001, **p < 0.01, *p < 0.05 vs. Aβ only

To confirm how RUR directly reduced Aβ accumulation in the 5xFAD mouse brain, we performed a Th T assay and dot blot after incubating Aβ peptides with RUR. NDGA, known to inhibit Aβ aggregation, was used as a positive control [26]. As a result, RUR incubated with Aβ_1-42_ monomer reduced aggregated Aβ compared to Aβ_1-42_ incubated alone (Fig. 7A). Moreover, to identify whether RUR affects Aβ oligomers, dot blotting was performed using the A11 antibody that reacts with Aβ oligomer. As a result, RUR incubated with Aβ_1-42_ monomer showed less Aβ_1-42_ oligomer aggregation than Aβ_1-42_ monomer incubated alone (Fig. 7B). Additionally, RUR incubated with Aβ_1-42_ oligomer eliminated this form of Aβ (Fig. 7C).

## Discussion

In this study, we investigated whether RUR treatment alleviated amyloid pathology in human APP and PSEN1 overexpressing transgenic mice. Our results demonstrate that the RUR attenuates memory loss in 5xFAD mice (Fig. 1). Additionally, RUR decreased the accumulation of Aβ protein including Aβ monomers, oligomers, and plaques in the brain of 5xFAD mouse (Figs. 2, 3). Additionally, this study showed that RUR alleviated the accumulation of LAMP1 and BACE1 within the DNs and tau hyperphosphorylation in the hippocampus (Figs. 4, 5, 6). Lastly, RUR inhibited Aβ aggregation and eliminated Aβ oligomers in vitro (Fig. 7).

The amyloid cascade hypothesis posits that Aβ accumulation in the brain is the primary cause of AD [33]. Under AD conditions, β-secretases induces APP endocytosis and sequentially cleaves APP together with γ-secretase within endosomes to produce monomeric Aβ, and this peptide is released into the extracellular space [34, 35]. The C-terminal of Aβ protein induces conformational changes from α-helix to β-sheet [36], leading to the aggregation of Aβ monomers into β-sheet rich oligomers, protofibrils, and fibrils [37]. In this study, RUR inhibited the further accumulation of Aβ aggregates in the brain of 5xFAD and suppressed the aggregation of Aβ peptides in vitro. These results suggest that RUR could directly target Aβ aggregates and protect against their impact on neurotoxicity.

BACE1, a transmembrane protein that plays a role in aspartyl protease activity, is commonly localized in the endosomes of brain neurons [38, 39]. Under healthy conditions, BACE1 is transported through endo-lysosomal organelles and is degraded by the lysosomal pathway [40]. However, as AD progresses, lysosomal dysfunction worsens and BACE1 accumulates in Aβ plaques in the dystrophic axons [41]. Lysosomal dysfunction causes BACE1 and APP to accumulate in immature lysosomes, upregulates β-site cleavage of APP, and increases Aβ production [38, 42]. Moreover, several studies have reported that under stressful conditions caused by pro-inflammatory cytokine, reactive oxygen species, and excitotoxicity, BACE1 expression increases and Aβ generation is accelerated [43]. Furthermore, Aβ aggregated forms upregulates protein levels of BACE1, indicating the loop of positive feedback [44]. According to several studies, the upregulation of BACE1 in the brain is associated with the development of AD. In this study, RUR treatment reduced the co-staining area of LAMP1 and BACE1, and these results showed that RUR could alleviate BACE1 accumulation within DNs around the Aβ deposit.

In the lysosomal degradation pathway, retrograde transport to the cell body is required for lysosomal maturation [45], and mature lysosomes degrade misfolded proteins, such as Aβ, that are delivered by endocytosis, autophagy, and phagocytosis [46]. However, in AD conditions, the excessive accumulation of Aβ causes lysosomal dysfunction, and these lysosomes accumulate within dystrophic axons adjacent to the Aβ burden [47]. Therefore, the intracellular transport of lysosomes to the cell body is disrupted by focal axonal swelling [48], resulting in abnormal lysosomal proteolysis, leading to the generation of DNs and lysosomal dysfunction [49]. Additionally, DNs contribute to the accumulation of Aβ plaques, which in turn leads to memory loss, cognitive impairment, neurodegeneration, and synaptic loss [16, 50]. Furthermore, recent studies have reported that mutations in APP and PSEN1 induce lysosomal dysfunction, disrupt axonal transport of lysosomes, and accumulate dysfunctional lysosomes in axons [51]. In this study, we identified that LAMP1-positive lysosomal vesicle accumulate within DNs around Aβ in the hippocampus of the 5xFAD mouse model, which overexpress human APP and PSEN1, and showed that RUR treatment could suppress the accumulation of dysfunctional lysosomes in the 5xFAD mouse.

Furthermore, we evaluated the effect of RUR treatment on tauopathy. Tau is a microtubule-associated protein that stabilizes the cellular cytoskeleton [52]. However, when tau proteins are highly phosphorylated, they dissociate from the cytoskeleton and accumulate in the brain, consequently inducing tauopathy, which is a pathological feature of AD [53]. In the brains of patients with AD, the levels of phosphorylated tau are 3–4 times higher than in the brains of healthy controls [54]. A previous study reported that the expression of phospho-tau (serine 202/threonine 205) in AD brains could predict the stage of tau pathology and these tau sites are phosphorylated by various kinases, such as GSK-3β and cyclin-dependent kinase 5 [55, 56]. Several studies have been reported that GSK-3β is excessively activated in the brains of AD patients, and the its activation is associated with memory impairment, synaptic loss, and neuroinflammation [57]. Moreover, GSK-3β activation induces Aβ generation and accumulation in the brain [58]. GSK-3β stimulates the mRNA expression of BACE1 thorough NF-κB pathway and induces APP cleavage by BACE1, resulting in Aβ deposits [32]. However, the phosphorylation of GSK-3β at serine 9 by Akt, which is known to play a role in cell survival and apoptosis, inhibits the activation of GSK-3β [59]. The dysfunction of the Akt/GSK-3β signaling pathway leads to excessive activation of GSK-3β, resulting in accumulation and aggregation of phosphorylated tau in the brain [60]. Several studies have shown that phosphorylated tau accumulates within DNs and blocks the trafficking of organelles in neurons [61, 62]. Moreover, another previous research showed that Aβ plaque itself can promote or facilitate tau aggregation and accumulation in the AD brain [63]. Abnormal aggregation and accumulation of phosphorylated tau are associated with synaptic loss, neuronal dysfunction, and memory impairment [64, 65]. Therefore, this study evaluated the effects of RUR treatment on tauopathy in the brains of 5xFAD mice by measuring the hyperphosphorylation of tau proteins at the molecular level. RUR treatment decreased the phosphorylation of tau at serine 202 and threonine 205 in the hippocampus of the 5xFAD mouse by regulation of Akt/GSK-3β signaling pathway. Therefore, our results suggest that RUR may alleviate the pathology of AD caused by tau hyperphosphorylation. However, since we used 5xFAD mice, which do not show neurofibrillary tangles [24], further studies would be required to demonstrate the ameliorative effect of RUR on tauopathy in a different model of AD such as P301S transgenic mice.

Collectively, this study showed that RUR was able to attenuate memory impairment and reduce Aβ accumulation, DNs formation, and phosphorylated ratio of tau in the hippocampus of 5xFAD mice. RUR treatment could suppress the generation of Aβ through downregulation of BACE1 within DNs, and directly prevent Aβ aggregation and degrade Aβ. Therefore, our results provide obvious evidence that RUR could effectively slow the progression of AD exacerbated by Aβ pathology, neuritic dystrophy, and hyperphosphorylated tau, and suggest that RUR could be a therapeutic supplements for the treatment of AD.

### Supplementary Information

Supplementary material 1.

## Data Availability

The datasets analysed during the current study are available from the corresponding author on reasonable request.

## Abbreviations

6E10: Anti-Aβ antibody
A11: Anti-oligomer antibody
AD: Alzheimer’s disease
APP: Amyloid precursor protein
Aβ: Amyloid-β
Akt: Protein kinase B
BACE1: β-Site amyloid precursor protein cleaving enzyme 1
CTF: C-terminal fragment
DNs: Dystrophic neurites
ECL: Enhanced chemiluminescence
GAPDH: Glyceraldehyde-3-phosphate dehydrogenase
HRP: Horseradish peroxidase
LAMP1: Lysosomal-associated membrane protein 1
NDGA: Nordihydroguaiaretic acid
PBS: Phosphate-buffered saline
RIPA: Radio-immunoprecipitation assay lysis buffer
PVDF: Polyvinylidene difluoride
RT: Room temperature
RUR: Rhei Undulati Rhizoma
sAPP: Soluble amyloid precursor protein
Th S: Thioflavin S
Th T: Thioflavin T
WT: Wild-type
