# Supplementary material for: Rhei Undulati Rhizoma attenuates memory decline and reduces amyloid-β induced neuritic dystrophy in 5xFAD mouse
Source: Chin Med. 2024 Jul 4;19:95. doi: 10.1186/s13020-024-00966-2 (PMC11223309; doi:10.1186/s13020-024-00966-2)
Supplement: Supplementary file 1 — Supplementary material 1. [file 13020_2024_966_MOESM1_ESM.docx]

**1. Materials and Methods**

*Morris water maze (MWM)*

The MWM test was performed as previously described. The maze used for the test included a round tank (45 cm height, 90 cm diameter) with four distinct visual cues on the wall. One hidden platform (29 cm height, 10 cm diameter) was placed in the center of a quadrant in the tank, which was conceptually divided. The tank was filled with water (24 ± 1°C) up to 1 cm above the hidden platform, and white opaque nontoxic paint was added to the water to hinder visibility. All mice were subjected to tree trials per day for 5 days. In each of the three trials, the animals were randomly placed at different starting positions equally spaced around the perimeter of the pool, and the mice were allowed 1 min to find the submerged platform. If the mouse did not mount the platform within 60 s, it was guided to the platform. The time to mount the platform was recorded as the latency for each trial. All mice were allowed to remain on the platform for 10 s before being returned to their cages. After 5 days of training, the probe trial, in which the platform was removed, was conducted. Each mouse was placed into the pool and allowed to swim for 1 min. All trials were recorded using a charge-coupled device camera connected to a video monitor and a computer.

**2. Supporting Figures**


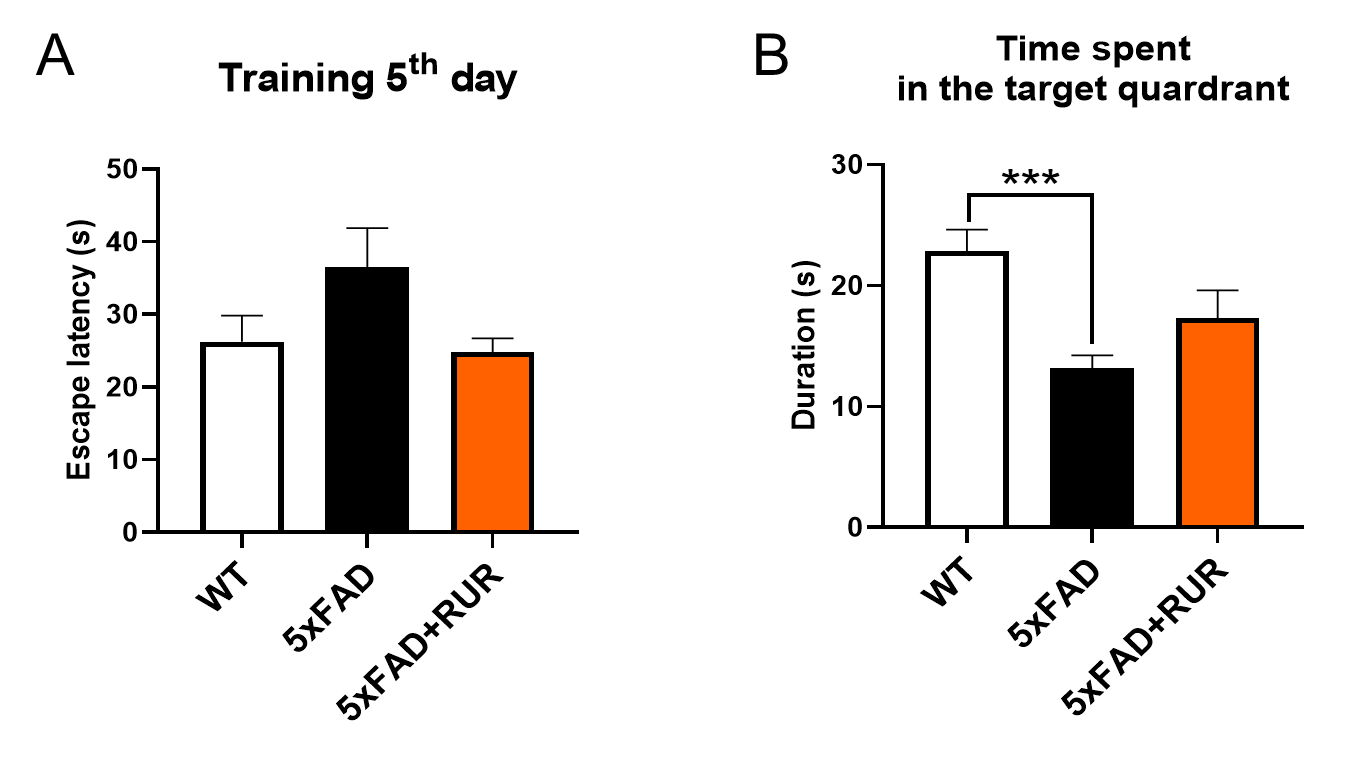


Supplementary Figure S1. Effects of Rhei Undulati Rhizoma (RUR) on memory impairment in 5xFAD mouse. Five-month-old WT and 5xFAD mice were administered vehicle or RUR (50 mg/kg) for 2 months. In the MWM, the escape latency on the 5^th^ day of training (A) and the time spent in the target quadrant on the probe trial were measured. The statistical analyses were performed using one-way analysis of variance (ANOVA), followed by Dunnett’s post hoc test. ****p* < 0.001 vs. 5xFAD group.

Supplementary Figure S2. Effects of RUR on the mRNA levels of β-secretase (BACE1) in the hippocampus of 5xFAD mouse (n = 5−6 per group). The mRNA level of BACE1 was quantified using quantitative reverse-transcription polymerase chain reaction (qRT-PCR). β-actin was used as an internal control. The statistical analyses were performed by unpaired Student’s t test. **p* < 0.05 vs. 5xFAD group.

Supplementary Table S1. Information of qRT-PCR primers used in this study.

| Gene | Primers | |
| --- | --- | --- |
| BACE1 | Forward | 5'-CCG GCG GGA GTG GTA TTA TGA AGT-3' |
|  | Reverse | 5'-GAT GGT GAT GCG GAA GGA CTG ATT-3' |
| β-actin | Forward | 5'-CTG GCA CCC AGC ACA ATG-3' |
|  | Reverse | 5'-GCC GAT CCA CAC GGA GTA CT-3' |
